# Glucagon and Its Receptors in the Mammalian Heart

**DOI:** 10.3390/ijms241612829

**Published:** 2023-08-15

**Authors:** Joachim Neumann, Britt Hofmann, Stefan Dhein, Ulrich Gergs

**Affiliations:** 1 Institute for Pharmacology and Toxicology, Medical Faculty, Martin Luther University Halle-Wittenberg, Magdeburger Straße 4, D-06097 Halle (Saale), Germany; ulrich.gergs@medizin.uni-halle.de; 2 Department of Cardiac Surgery, Mid-German Heart Center, University Hospital Halle, Ernst Grube Straße 40, D-06097 Halle (Saale), Germany; britt.hofmann@uk-halle.de; 3 Rudolf-Boehm Institut für Pharmakologie und Toxikologie, Universität Leipzig, Härtelstraße 16-18, D-04107 Leipzig, Germany; stefan.dhein@medizin.uni-leipzig.de

**Keywords:** glucagon, glucagon receptor, human heart, mouse heart

## Abstract

Glucagon exerts effects on the mammalian heart. These effects include alterations in the force of contraction, beating rate, and changes in the cardiac conduction system axis. The cardiac effects of glucagon vary according to species, region, age, and concomitant disease. Depending on the species and region studied, the contractile effects of glucagon can be robust, modest, or even absent. Glucagon is detected in the mammalian heart and might act with an autocrine or paracrine effect on the cardiac glucagon receptors. The glucagon levels in the blood and glucagon receptor levels in the heart can change with disease or simultaneous drug application. Glucagon might signal via the glucagon receptors but, albeit less potently, glucagon might also signal via glucagon-like-peptide-1-receptors (GLP1-receptors). Glucagon receptors signal in a species- and region-dependent fashion. Small molecules or antibodies act as antagonists to glucagon receptors, which may become an additional treatment option for diabetes mellitus. Hence, a novel review of the role of glucagon and the glucagon receptors in the mammalian heart, with an eye on the mouse and human heart, appears relevant. Mouse hearts are addressed here because they can be easily genetically modified to generate mice that may serve as models for better studying the human glucagon receptor.

## 1. Introduction

When entering the term “glucagon” into PubMed, one records (as of 24 May 2023) about 53,000 hits. The present review focused on a small part of this large research effort on glucagon. We are merely dealing with the contractile role of glucagon in the heart.

Glucagon is mainly formed in α-cells in the islets of the Langerhans within the human pancreas [1]. In the human liver, but to a lesser extent in the heart, glucagon augments gluconeogenesis and glycogenolysis. Therefore, glucagon acts as a functional antagonist of insulin: insulin decreases the blood concentrations of glucose, and glucagon increases the blood glucose levels.

**Figure 1:**
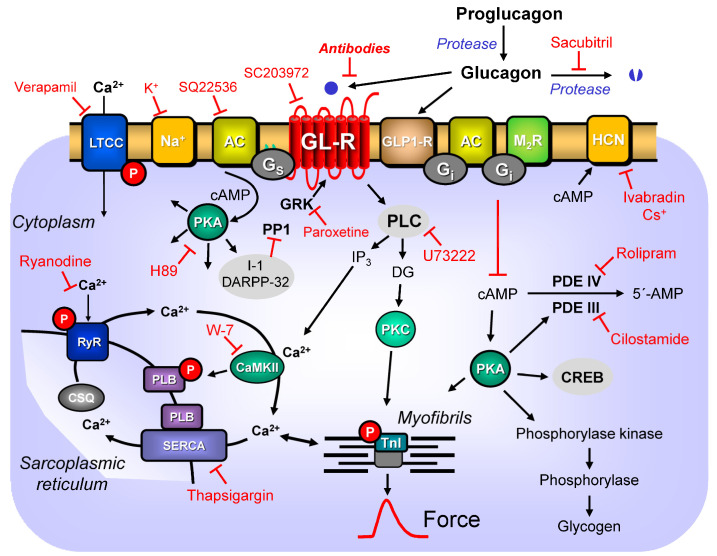
Potential mechanism(s) of action of glucagon in the human and mouse cardiomyocytes. Stimulation of the activity of the glucagon receptor (GL-R, blocked by antibodies or SC203972) by endogenous glucagon leads via stimulatory GTP-binding proteins (G_s_) to an increase of adenylyl cyclase (AC) activity (inhibitable by SQ22536). AC increases the formation of 3′,5′-cyclic adenosine mono phosphate (cAMP) that stimulates cAMP-protein kinases (PKAs, inhibitable by H89). PKAs phosphorylate (red P) and thus activate phospholamban (PLB) at the amino acid serine 16, the inhibitory subunit of troponin (TnI), the ryanodine receptor (RYR), the L-type calcium channel (LTCC, inhibitable by verapamil), cAMP response element-binding proteins (CREB), or phosphorylase kinase (which then phosphorylates and activates phosphorylase to cleave glycogen). PKA also phosphorylates the so-called phosphatase inhibitors such as inhibitor 1 (I1) or dopamine- and cAMP-regulated neuronal phosphoprotein (DARPP32) that then inhibit the activity of serine/threonine protein phosphatase 1 (PP1). PLB is also phosphorylated by calcium-calmodulin-dependent protein kinases (CaMKII: inhibited by W-7) on amino acid threonine 17. cAMP can directly activate the hyperpolarization-activated cyclic nucleotide-gated channels (HCN) in the sinus node (inhibited by ivabradin or cesium ions (Cs^+^)). The formed cAMP can be degraded to inactive 5′-adenosine mono phosphate (5′-AMP) by phosphodiesterases (PDE III: inhibited by cilostamide, PDE IV: inhibited by rolipram). Calcium cations (Ca^2+^) are stored on calsequestrin (CSQ) in the sarcoplasmic reticulum (SR) and are released via RYR (inhibitable by ryanodine) from the SR. The released Ca^2+^ bind to thin myofilaments and as a result systolic force is augmented. In cardiac diastole, concentrations of Ca^2+^ fall because Ca^2+^ are pumped into the SR via the SR-calcium ATPase (SERCA, inhibitable by thapsigargin). GL-R can be inactivated by G-protein-dependent protein kinases (GKR: inhibited by paroxetine). GL-R can activate phospholipase C (inhibited by U73222). Cardiac sodium channels (Na^+^) in the sarcolemma can be functionally inhibited by potassium ions (K^+^) that lead to partial depolarization. Proglucagon can be cleaved to glucagon. Glucagon is degraded by another protease (inhibited by sacubitril). Glucagon can also activate glucagon-like-protein-1-receptors (GLP1-R). Acetylcholine can activate M_2_-muscarinic receptors and thereby, via GTP-binding inhibitory proteins (Gi), reduce the activity of AC: see text for further details.

Glucagon was one of the first stimuli identified as an adenosine-3′,5′-cyclic monophosphate (cAMP, Figure 1)-increasing agent (liver: [2]). The positive inotropic effects of glucagon in the heart were initially thought to result from the release of stored noradrenaline and subsequent stimulation of the β-adrenoceptors in the heart [3]. However, these positive inotropic effects and cAMP-increasing effects of glucagon in the heart were later shown not to be blocked by the β-adrenoceptor antagonist propranolol (dog: [4], cat, and human: [5]), and thus it is regarded as being mediated by a receptor of its own: the glucagon receptor. Yet, glucagon can also activate another receptor called the glucagon-like protein-1-receptor (GLP1-R, Figure 1). Indeed, glucagon-like peptide-1-receptors and related so-called glucagon-like protein-2-receptor are also expressed and functional in the heart [6,7]. Glucagon is not an agonist to the glucagon-like protein-2 receptor [8]. Accordingly, the glucagon-like protein-2-receptor will not be discussed in this review.

## 2. Glucagon Receptor

Initially, the rat glucagon receptor was cloned in the year 1993, thirty years ago [9]. The human glucagon receptor was cloned in 1994 and displayed an 82% protein sequence identity with the rat glucagon receptor [10]. The binding affinity of glucagon to the human glucagon receptor was reported to be 5 nano mole per liter (nM) of glucagon [10]. A wealth of data has defined the three-dimensional structure of the glucagon receptor and how the agonist glucagon or its analogs bind to this receptor [11,12,13,14]. This issue has been discussed elsewhere and is not the focus of the current review [15].

That the glucagon receptor is present in the mammalian heart, specifically in the rat heart, was already reported in the first cloning papers [9,16]. Using an RNase protection assay, Hansen et al. [17] quantified the messenger ribonucleotides (mRNA) for glucagon in a rat heart. In their hands, the mRNA expression of the glucagon receptor in the heart was about 50% of the mRNA expression of the glucagon receptor in the rat liver [17]. This relatively strong expression may indicate an essential function of glucagon receptors in the heart. Later, the glucagon receptor was found in mRNA levels in the mouse heart [18]. The glucagon receptor as mRNA was highly expressed in the mouse’s right atrium, but very lowly expressed in the left atrium or cardiac ventricle [19]. One would predict that the contractile function of the glucagon receptor would follow its regional expression, which is, to some extent, apparently the case (see below for the rat heart). On a protein level, using radioactive glucagon and autoradiography, glucagon receptors have also been detected in the mouse heart, suggesting a possible functional role of the glucagon receptor also in the mouse heart [20].

The human glucagon receptor comprises only 477 amino acids, whereas the mouse and rat glucagon receptors comprise 485 amino acids [16,21]. In human glucagon-receptor-transfected cells, glucagon stimulates adenylyl cyclase (AC) very potently, with a half maximally stimulatory concentration (EC_50_-values) of 10 pico mole per liter (10 pM) [21]. This high potency of glucagon in the stimulation of the glucagon receptor is important to keep in mind, because this potency is much higher than the concentration of glucagon required to raise the beating rate or force of contraction in the mammalian heart.

The human glucagon receptor is located on chromosome 17 at 17q25 [21]. It may be relevant that the protein sequence of the human glucagon receptor is not only shorter than that of the mouse glucagon receptor (see above), but there is only about an 80% protein sequence identity between the human and mouse glucagon receptors [22]. This may translate into species differences in glucagon receptor function. Therefore, arguments can be made that the normal mouse heart might not be the best model for understanding the human cardiac glucagon receptor. This concern for the species differences of the glucagon receptor has prompted the development of transgenic mouse models, where the human receptor is expressed instead of the endogenous mouse receptor in the mouse heart and functional differences are searched for (see below).

The mRNA of the glucagon receptor has been detected in all regions of the human heart [7]. However, the expression of the human glucagon receptor at the mRNA level is very heterogeneous with respect to the region of the heart, but also with respect to the patient being studied. For instance, in one study, no expression of glucagon receptors was found using reverse transcriptase polymerase chain reaction (RT-PCR) in the left human ventricle, and in only 2 of 15 different patient samples was the expression of the glucagon receptor in the right ventricle noted with RT-PCR, while in 3 of 15 patient samples with RT-PCR, the expression of the glucagon receptor was noted in the right atrium. The glucagon receptor was detectable in 1 of 15 patient samples in the left atrium [7]. Other researchers, using different patients and slightly different methodologies, failed to detect any expression at the mRNA level or protein level (Western blotting) of the glucagon receptor in samples from the heart (left atrium, right atrium, left ventricle, right ventricle, and sinus node, [23]): they studied samples from a total of ten patients; this included diseased patients (e.g., hypertrophic obstructive cardiomyopathy or autoimmune myocarditis), but also tissue from donors that had died from accidents (remarkably, only two donors did not take any drugs, which might have affected the glucagon receptor expression in the heart). The donor tissues were in asystole for a mean period of 83 min. This might have led to degradation of the mRNA for the glucagon receptor [23]. Likewise, others have failed to detect mRNA of the glucagon receptor in the human heart [24]. They studied cardiac tissue from over 100 donors [24]. They used atrial tissue (left or right atrium was not specified) and left ventricular tissue from patients [24]. Moreover, these cardiac samples were studied post mortem [24]. Hence, one could argue that the mRNA for the glucagon receptor from the cardiac tissue may have been degraded during processing and, therefore, the mRNAs for the glucagon receptor in the hearts were not detected [23,24]. This assumption is not very far fetched: the glucagon receptor has been functionally found in human coronary arteries (Table 1). Hence, when mRNA is prepared from the whole human heart, at least these vascular glucagon receptors should have been detectable in the mRNA prepared from the whole human heart samples. As this was not the case in these studies [23,24], the possible degradation of the mRNA of the glucagon receptor cannot be completely ruled out as a limitation of these studies [23,24].

If these results [7,23,24] are representative and typical of the human heart and not just chance findings, the published contractile data are hard to reconcile with these mRNA expression data of the human cardiac glucagon receptor: in most samples from failing and non-failing ventricular muscle strips, glucagon exerted positive inotropic effects (Table 1). In the study by Baiio et al. [7], no contractile function of glucagon was measured in samples that were analyzed for the mRNA expression of the glucagon receptor in human heart tissue [7]. One would have predicted in their tissues [7] no positive inotropic effects of the glucagon in the atrial and ventricular muscle strips in which the mRNA of the glucagon receptor was measured, and no inotropic effects of glucagon in the samples where the mRNA for the glucagon receptor was below the detection limit.

Here, one must remember that the glucagon given to patients or animals might not only stimulate the glucagon receptor, but also the related GLP-1 receptor. While GLP-1 binds to the human GLP-1 receptor with an affinity of around 5 nM, glucagon is about 100 times less potent at binding to the human GLP-1 receptor [73].

In many studies (Table 1), 1–10 micro mole per liter (μM) of glucagon was given in an organ bath to stimulate the force of contraction in human cardiac muscle preparations. A total of 1 µM of glucagon should easily stimulate both the glucagon and GLP-1 receptors in the human heart (Table 2).

The glucagon-stimulated generation of cAMP has similar EC_50_-values (half maximum concentrations of stimulation) in mouse livers as those in humanized mouse livers (40 nM glucagon and 13 nM glucagon, respectively, [91]). In contrast, in human liver membranes, glucagon-stimulated cAMP formation has an affinity of 6 nM [92]. These values are somewhat higher than the physiological levels of glucagon in the blood. However, from these results, one would predict similar EC_50_ values (that is, 13 nM to 40 nM) for the positive inotropic and positive chronotropic effects of the glucagon in muscle strips from experimental animals or human muscle strips if the human cardiac glucagon receptor mediates the contractile effects of the glucagon in the human heart. However, this is not the case under all conditions, and this issue will be addressed in more depth below.

More recently, systematic efforts have been made to identify the glucagon receptor with a battery of antibodies. Using tissue from glucagon receptor knockout (KO) mice as a negative control, two out of twelve commercially available receptor antibodies were identified that could detect the glucagon receptor in Western blots at about 55 kDa [24]. Only one of the twelve antibodies was specific in fixed liver sections [24]. With this specific antibody in their hands, researchers could identify the glucagon receptor in the immunohistochemistry of the mouse heart on cardiomyocytes [24]. Apparently, they did not study with this specific antibody the expression of the glucagon receptors in the human heart [24], which would have been interesting given the discrepancies in the mRNA expression data for the glucagon receptors in the human heart, as discussed above.

There is speculation in the literature that not all cardiac effects of glucagon can be explained by its cognate glucagon receptor or by its cross-reactivity to the glucagon-like peptide 1 receptor. Still, there might be an unknown orphan glucagon receptor in the human genome [93]. Hence, this chapter might not yet be closed.

Autoradiography with radioactively labeled glucagon has failed to detect a specific signal in the human heart [24]. This has been discussed as to possibly indicate that the glucagon receptor is lacking in the human heart, because radioactive glucagon is expected to bind to the glucagon receptor and thus induce a signal in autoradiography, indicative of the presence of the glucagon receptor [24]. This lack of signal in autoradiography might mean that the glucagon receptors are not present on the surface of cardiomyocytes, but are mainly in the cytosol, where they cannot react with radioactively labeled glucagon, but could react with an antibody [24].

As mentioned above, glucagon has about 140 times less affinity for glucagon-like peptide receptors (about 130 nM) than the specific agonists of these receptors. GLP-1 receptor expression as mRNA was three times more abundant in the human atrium than in the ventricle or cardiomyocytes from the human left ventricle [94]. In isolated, electrically driven atrial muscle strip preparations from ten patients, a GLP-1 receptor agonist (6 nM and higher of exenatide, a selective GLP-1 receptor agonist that does not activate the glucagon receptors, Table 3) concentration- and time-dependently raised the force of contraction [94]. It is interesting to note, but not readily understood, that only in 2 of 14 ventricular muscle strips from non-failing human (donor) hearts, did exenatide exert a positive inotropic effect. In contrast, all the human atrial and human ventricular samples in this study expressed the mRNA of the GLP-1 receptors [94]. GLP-1 receptor expression as a protein in the human heart was not reported [94]. Therefore, perhaps in the ventricular tissue, the protein expression of GLP-1 is usually less in the human ventricle than that in the human atrium, which may explain this discrepancy in function [94]. If we assume the lowest estimate of the EC_50_-value of 6 nM for the GLP-1 receptor, and given that glucagon is 100 times less potent at the GLP-1 receptor than exenatide is, the lowest EC_50_ estimate of glucagon at the GLP-1 receptor would be 600 nM. Therefore, a positive inotropic effect occurring at around 600 nM to 1 μM of glucagon (reported in many studies on cardiac effects on the force of contraction in human hearts, shown in Table 1) could mean that these effects are mediated by glucagon acting through the GLP-1 receptors. This should be studied directly by repeating such experiments. One could study in the same muscle strips exenatide and glucagon in head-to-head comparison. One could also include GLP1-receptor antagonists and, in comparison, also antagonists selective for glucagon receptors in such contraction experiments (possible antagonists have been selected in Table 3).

However, the published data would be consistent with the assumption that, at least in the non-failing human heart, the positive inotropic effect of glucagon is mediated, in all likelihood, by two receptors. At low glucagon concentrations, glucagon would stimulate the glucagon receptors in human atrial and ventricular preparations. At higher glucagon concentrations, glucagon would stimulate the GLP-1 receptors. When glucagon stimulates the glucagon receptor, as well as when glucagon stimulates the GLP-1 receptor, an increase in the cAMP concentrations in cardiomyocytes would follow, and this would increase the force of contraction in any case (Figure 1).

Interestingly, as heart failure (detected clinically, patients aged from 18 years to 62 years) increased, the positive inotropic effect was weaker and was even absent in papillary muscle strips from patients with end-stage heart failure, and, in their samples, glucagon failed to increase the activity of AC [60].

As animal models, to better understand the role of the glucagon receptors, mice with a generalized knockout of the glucagon receptor or heart-specific knockdown of the glucagon receptor have been generated [18]; they have a cardiovascular phenotype [18] that will be discussed below. However, these mice would also be helpful in the future to study the inotropic and chronotropic effects of glucagon. One would predict that glucagon should be unable to increase the force of contraction in the atrium and/or ventricle of mice with a knockout of the glucagon receptor.

## 3. Glucagon Receptor Regulation

In principle, the expression of the glucagon receptor can be regulated, and thereby the function of glucagon can be changed in the heart. In isolated, cultivated adult rat hepatocytes after 24 h, the glucagon receptor (at the mRNA and protein level) is down-regulated in the presence of glucose and upregulated in the presence of cAMP-increasing agents, acting via the stimulation of adenylyl cyclase (AC) through forskolin, the activation of the glucagon receptor through glucagon, or the inhibition of cAMP degradation (using a phosphodiesterase (PDE) inhibitor, 3–isobutyl-1-methyl-xanthine) and the stimulation of the β-adrenoceptor (by isoprenaline, [104]). If we assume that the glucagon receptor stimulates AC, then the measurement of AC is a surrogate parameter of glucagon-receptor–protein expression. Under this limitation, one can try to interpret older data generated before cloning the glucagon receptor was described.

Experimental hypothyroidism reduces the efficacy (but not the potency) of glucagon to stimulate AC (Table 2) in the hearts of rats, conceivably via a reduction in the density of the glucagon receptors [105]. Chronic β-adrenergic stimulation with a parenteral injection of living rats with isoprenaline likewise reduces the efficacy (but not potency) of glucagon to increase the activity of cardiac AC [106]. In the hearts of obese or hypertensive rats, the efficacy of glucagon to stimulate the activity of AC is reduced. In the dog heart, glucagon is more effective in stimulating the activity of AC in the ventricle than the atrium [81]. In the monkey atrium, glucagon fails to activate AC [81]. In human atrial or ventricular preparations, 10 μM of glucagon increases the activity of AC [107]. The problem is that very high concentrations of glucagon are required to detect an increase in AC activity.

Nevertheless, artifacts should be ruled out. Hence, there is a clear need to also measure in protein levels the expressional changes alluded to above for the glucagon receptor, for example, in hypothyroidism or after prolonged β-adrenergic stimulation, to confirm or refute this earlier work.

Like other heptahelical receptors, the glucagon receptor exhibits homologous desensitization due to receptor downregulation. At least this could be concluded when rats were treated with injections of 0.5 milli gram (mg) of glucagon/150–200 g (g, body weight) over 10 days [108]. The density of the signal (the binding of the membranes from the rat liver to the radioactive glucagon) declined [108]. However, PCR or better Western blot data in the heart would also be interesting under these conditions to confirm this reduced glucagon receptor expression over time. In a guinea pig left atrium, 1 μM of glucagon increased the force and cAMP levels, and both these parameters were desensitized by a 15 min pre-treatment with 2 μM of glucagon [43]. Similarly, the desensitization of glucagon in cardiac contractility is well known [3,44,45]. In mice in vivo, glucagon induced tachycardia [65]. In the liver, the desensitization of AC by glucagon has been convincingly demonstrated [109]. Desensitization for all G-protein-coupled receptors involves isoforms of the GTP-binding protein receptor kinase (GRK), protein kinase C, and a cAMP-dependent protein kinase (PKA, [110], Figure 1).

Glucagon can lead to the phosphorylation of the glucagon receptor in Chinese hamster ovary cells transfected with this receptor [111]. One would predict this to occur in the human heart, but this has not yet been reported. For receptor regulation, it is essential to know that glucagon receptors can be ubiquitylated [112,113,114]. This ubiquitination is involved in the internalization of the glucagon receptor upon agonist occupation. In addition, ubiquitination seems to drive biased agonism; it can lead to the stimulation of the non-canonical pathway comprising β-arrestin and MAP kinases [113]. The expression of the glucagon receptor in a transfected cell line could be reduced by antidiabetic drugs, namely thiazolidinediones [115]. One could speculate that this mechanism might be active in the heart. This is consistent with the information available for the promoter of the glucagon receptor, which contains an element involved in the mechanism of action of thiazolidinediones [116]. In the rat glucagon receptor gene, a glucose response element reduces the transcription of the glucagon receptor and, thus, its expression [117]. The promoter region of the human glucagon receptor has been studied [116]. This is relevant for the downregulation of the glucagon receptor. There is evidence for a cAMP-mediated downregulation of promoter transcription in cell culture studies [116]. This would, for instance, explain why glucagon can reduce the expression of the glucagon receptor by raising the cAMP level. This is a protective mechanism against deleterious increases in the cellular (in our case, cardiac) cAMP levels by auto-inhibiting the action of glucagon. Similarly, glucose autoinhibition exists. In liver cells in culture, high glucose concentrations reduce the efficacy of the glucagon receptors to increase glucose concentrations further [100]. Whether this holds true in the heart remains to be studied.

## 4. Glucagon Receptor Agonists and Antagonists

As already mentioned above, glucagon has about 140 times less affinity for glucagon-like peptide receptors (about 130 nM) than specific agonists at these receptors (review: [9]). GLP-1-receptor expression as mRNA is three times more abundant in the human atrium than the human ventricle or cardiomyocytes from the left ventricle [94].

Here, species differences occur again, making it challenging to translate animal data directly to patients. In rat cardiomyocytes, glucagon-like peptide-1 increased cAMP levels but reduced contractility. This was accompanied by a reduced pH in the cardiomyocytes and a subsequent desensitization of the myofilament to calcium [118] (review: [119]). In contrast, the stimulation of GLP-1 receptors increased the force of contraction in human atrial preparations [94]. Peptides similar to glucagon can be used as agonists and antagonists at the glucagon receptors [119,120]. Taking a pharmacokinetic approach, non-peptide antagonists at the glucagon receptor have been developed that are per-orally available and might play a role in treating diabetes [12,119]. Some authors have claimed that glucagon fails to pass through the blood–brain barrier [121]. Hence, glucagon infused to treat the heart should not have direct side effects in the brain. However, this view has been challenged in recent years; some have claimed that glucagon can pass into the brain and exert physiological effects in the brain [122].

There has been some success in devising drugs comprising glucagon, a linker, and triiodothyronine. This bifunctional agonist was helpful in a mouse model of diabetes for preserving cardiovascular function [123]. Peptide fragments of glucagon or mutated glucagon stimulate the activity of AC in a cell culture system, usually with a potency and effectivity less than those of native glucagon. As such, these derivatives of glucagon are partial agonists, and thus, they are also antagonists at the glucagon receptor [82]. Another classification of glucagon receptor antagonists is to divide them based on their chemistry into small organic molecules (not protease-sensitive peptides), antibodies, or antisense ribonucleic acid (RNA) [124]. Theoretically, antisense RNA could also be encapsulated in a virus. Antagonists were initially designed to reduce the blood glucose levels (mice and monkeys: [125]).

Peptide analogs of glucagon have been radioactively labeled and used as tracers to study the glucagon receptor occupation in the living body [126]. Like insulin, glucagon can precipitate in solution or stick to the vessel wall. Hence, some have recommended the addition of bovine serum albumin and an acidic pH for its storage [127]. Others have developed a mutated glucagon called dasiglucagon, which contains seven amino acid mutations compared to glucagon and does not cause fibrils to form in aqueous solutions [113].

## 5. Pharmacokinetics of Glucagon

Being a peptide, glucagon as a drug cannot be given per os: one needs to bypass the gastrointestinal tract. Therefore, in cardiovascular studies on living animals or patients, glucagon is given parenterally [128], usually intravenously. In clinical practice, however, glucagon is approved for application intramuscularly, subcutaneously, or intranasally [113]. In these studies, 1 to 5 mg dosages of insulin are used, depending on whether children or adults are being treated [113]. Other parenteral routes of application have been used in patients or at least as proof of principle in animal studies, including subcutaneously [129] and intranasally [130].

The half-life of glucagon in healthy humans’ plasma is about 5 min after intravenous or intranasal application and 30 min after intramuscular application [131]. The bioavailability of intranasal glucagon is 30% of intramuscular application [131].

Nevertheless, once it became clear that glucagon works on glucagon receptors, small organic molecules, stable against intestinal metabolism, were developed to stimulate the glucagon receptor(s). As a next step, small organic molecules were found, typically containing amide groups that are only slowly broken down by human proteases and that inhibit the glucagon receptor(s) when given perorally [95,99]. These drugs are currently being intensely studied to develop marketable drugs for weight loss in obesity [132]. Hence, knowing the cardiac side effects of such anti-obesity drugs is essential. Another clinical application of glucagon can be functional studies on the upper gastrointestinal tract in X-ray procedures [133]. Glucagon injected subcutaneously by a patient’s relatives is sometimes used to treat hypoglycemic comas in diabetes mellitus 1 [134]. In that study [134], as expected, an intravenous injection of 1 mg of glucagon led to higher plasma concentrations than a subcutaneous or intramuscular injection of 1 mg of glucagon. Within minutes, the 1 mg of glucagon intravenously reached a peak concentration of about 30 nM (105 pg/mL) glucagon that rapidly declined thereafter.

From such studies, one can conclude that no relevant direct cardiac effects are expected under the standard therapeutic dosing of glucagon (1 mg) (see below). When glucagon is given subcutaneously in clinical studies, there is a dose-dependent increase in the plasma levels of glucagon, with peak values reaching about 1 nM of glucagon in about 20 min in a typical study [135]. When 150 micrograms (µg) of glucagon were given to patients with type 1 diabetes, peak blood glucagon levels of about 40 pM were reached, increasing their blood glucose values [136]. In another study, 1 mg of glucagon given subcutaneously via the abdomen led to plasma glucagon levels of around 1 nM [135]. Infusion or bolus intravenously in healthy volunteers at 50 micro grams (μg) of glucagon per kilogram (kg) of body weight led to an increase in glucagon from 7 pM to 82 nM or 107 nM. This high glucagon dosage led to nausea, even in the presence of granisetron (8 mg) in 70% of the participants [63].

## 6. Formation and Degradation of Glucagon

Glucagon is formed through cleavage by a protease called proconvertase 2 (Figure 1) from a precursor protein called proglucagon [6,137,138]. Proconvertase 2 is primarily expressed in the pancreas and this regional expression explains the high levels of glucagon in the pancreas [139]. Relevantly, the expression of this proconvertase 2 is also measured in the heart, albeit in the rat heart, and only in the intracardiac ganglia [140,141]. Hence, glucagon can be produced in the heart and can act in a paracrine or autocrine fashion.

Mice with a knockout (KO) of proconvertase 2 [141] have been described. In these mice, their glucagon levels were nearly undetectable [142]. These data [142] strongly support the view that in vivo glucagon is formed via the action of proconvertase 2. Still, other proteases might also catalyze glucagon formation, but to a much lesser extent than proconvertase 2 [141].

One may speculate that the concentration of glucagon formed in the heart is altered in heart failure (in analogy to, for instance, atrial natriuretic peptide). In blood from healthy adult subjects, 10–20 pM of glucagon was reported (review on the normal plasma glucagon concentrations in several species and vessels: [143]). In an early study on heart failure, much higher values than these normal plasma glucagon concentrations of 10–20 pM, namely 260 pg/mL of glucagon, were measured in patients (about 700 pM, [143]). Thus, elevated glucagon levels in the blood may accompany heart failure. In contrast, a later study failed to detect any increase in the plasma glucagon in patients with heart failure [144]. However, these patients were clinically different (from [143]), and different drug treatments (against their heart failure) were used [144]. A more recent, larger study (140 heart failure patients and 20 healthy controls) measured a significant elevation in the blood glucagon concentrations in patients suffering from systolic heart failure compared to appropriate control subjects [145].

Glucagon is degraded by several enzymes (Figure 1). Of potential clinical relevance is the degrading enzyme neprilysin (neutral endopeptidase 24.11). This neprilysin might be relevant because sacubitril can inhibit neprilysin [146,147]. Sacubitril is currently used in drug combinations to treat heart failure patients. In mice with a KO of neprilysin, their peripheral blood pressure was lower than that in control mice, consistent with glucagon-mediated arterial peripheral vasodilatation [148,149]. One might speculate that some of the beneficial effects of scubitiril in heart failure patients might be due to elevated plasma glucagon levels.

In cardiac cells and human plasma, proteases other than neprilysin can cleave glucagon and form active peptides of glucagon [150,151]. One of these peptides, called “mini-glucagon” (comprising the amino acids 19–29 of glucagon, Figure 1), may potentiate the effects of glucagon, at least in the rat heart [75,152]. Whether mini-glucagon potentiates the effects of glucagon in the human heart has never been published. An alternative enzymatic cleavage of proglucagon leads to glucagon-like protein-1, which can inhibit the release of glucagon from the pancreas (Figure 1). Proglucagon is not only expressed in the pancreas, but also in the heart (mouse: [153]). Hence, glucagon might be formed in the heart by the cleavage of proglucagon.

## 7. Signal Transduction

As mentioned in the introduction, glucagon can stimulate the activity of AC (Figure 1), namely in broken-cell preparations from the human atrium via stimulatory guanosine triphosphate (GTP)-binding proteins. However, glucagon can also inhibit the activity of cardiac adenylate cyclases via inhibitory GTP-binding proteins in membrane preparations [78,154]. It is uncertain whether the glucagon receptors can act in vivo via inhibitory G-proteins. In hearts from pertussis toxin-treated rats (in which inhibitory G-proteins were functionally inactivated by this pertussis toxin), one would have expected that glucagon would be more potent to raise the force of contraction, but this was not the case [67]. The potency of glucagon to increase the force of contraction was not different compared to the control conditions [67]. However, comparable studies on the human heart are missing. In principle, it is feasible to isolate human cardiomyocytes, treat them with pertussis toxin [155], and then apply glucagon and measure contractility. However, this is speculation.

In frog ventricular cardiomyocytes, glucagon does not stimulate the activity of AC in membranes [42]. However, opposing views were later published when a frog (Rana tigrina rugulosa) glucagon receptor was transfected into cells (Chinese hamster ovary cells). In these transfected cells, the glucagon raised the cAMP levels [156]. One might speculate that the coupling of the frog glucagon receptor to AC is cell type specific and the necessary proteins for the glucagon receptors coupling to AC are missing in frog cardiomyocytes. In contrast, glucagon inhibited the activity of PDE, thus increasing the cAMP content in the frog heart [42,77]. This formed cAMP activates PKA. PKA is expected to phosphorylate and activate the L-type calcium channel (LTCC). Indeed, in frog and rat ventricular cardiomyocytes, glucagon increases the current through the LTCC [42]. Moreover, PKA is expected to phosphorylate phospholamban and the troponin inhibitor (TnI). This would explain why glucagon shortened the total contraction time in anesthetized dogs [30] and the time of relaxation in guinea pig left atria and isolated, perfused rat hearts [45,58]. Indeed, glucagon (0.2 μM) increased the force and phosphorylation of TnI in isolated rat hearts [83], which facilitated cardiac relaxation under glucagon treatment. In canine cardiac membranes, glucagon increases the uptake of calcium ions. It altered the loss of calcium ions from isolated, perfused rat hearts [157,158], which is consistent with a stimulatory action on phospholamban phosphorylation (Figure 1). However, in contrast to TnI phosphorylation [83], glucagon inducing phospholamban phosphorylation in hearts has apparently never been reported. This would be an important step in signal transduction, because the phosphorylation of phospholamban can contribute to enhancing relaxation rates and increasing the contraction rates in hearts. Using aequorin, glucagon increased divalent calcium ion(s) (Ca^2+^) transients in rat cardiac preparations [159]. Glucagon shortened the time to peak force and to peak Ca^2+^ transients under these conditions [159]. Likewise, in chicken embryonic ventricular cardiomyocytes, glucagon could increase Ca^2+^ transients [75]. Glucagon can also increase the uptake of Ca^2+^ into rat cardiac mitochondria, which might serve a physiological function [160]. Moreover, glucagon (0.25 μM) increased the pyruvate dehydrogenase activity in the isolated, perfused heart, which was also explained by increased calcium ion activity in the heart’s mitochondria and is involved in glucose metabolism [161].

Consistent with the formation of cAMP after glucagon stimulation in rats, the efficacy or potency of glucagon in the rat ventricle to raise contractility was increased by adding theophylline (an unselective PDE inhibitor [69]; rolipram a PDE4 inhibitor, [84] or cilostamide, a PDE 3 inhibitor, [85]). Consistent with the lack of a glucagon-induced positive inotropic effect in the rabbit heart, glucagon did not increase the AC activity in preparations from rabbit hearts [66].

Interestingly, low concentrations of glucagon (100 pM, too low to increase the force of contraction) activate protein kinase B and phosphoinositide-3-kinase, at least in isolated, perfused rat hearts [68]. This indicates that low glucagon concentrations (below the threshold concentration of glucagon required to increase the force of contraction) trigger signal transduction mechanisms other than higher glucagon concentrations, at least in the rat heart. Thus, glucagon receptors in the heart may switch from one signal transduction system to the next. These data are also consistent with the assumption that only artificially elevated glucagon concentrations elevate the force of contraction. In contrast, physiologically low glucagon concentrations may serve essential metabolic functions in the heart.

In rat ventricular cardiomyocytes, the effects of isoprenaline and glucagon on the increase in the current through the LTCC are not additive, suggesting that both drugs finally use the same pathways, namely, the stimulation of AC, cAMP generation, activation of PKA, and phosphorylation of the LTCC, to increase the force of contraction [42]. Consistent with the stimulatory role of glucagon on the activity of AC in the heart, compounds that inhibit the activity of AC are expected to reduce the positive inotropic effects of glucagon. This category includes acetylcholine, which is well known to inhibit AC under certain conditions. Indeed, acetylcholine attenuated the positive inotropic effect of glucagon in canine papillary muscles (8 µg/mL) [16]. Moreover, 3 μM of glucagon increased the cAMP levels in canine ventricular muscle preparations, and this increase in cAMP was reduced by an additionally applied 3 μM of carbachol, an M-cholinoceptor agonist [31].

In dogs, glucagon exerts a positive chronotropic effect if perfused in the sinus node artery and increases the conduction velocity through the AV node [31]. Moreover, glucagon increased the force of contraction in the isolated canine atrium [3,31]. There are conflicting data suggesting species differences in the response of cGMP to glucagon. In the canine ventricle, 3 µM of glucagon elevated cGMP, which was further increased by 3 μM of carbachol [31]. This elevation of cGMP might follow the inhibition of PDE by glucagon. Alternatively, glucagon may stimulate the activity of guanylyl cyclase, directly or indirectly (via nitric oxide production). In contrast, in frog and rat hearts, glucagon failed to increase the cGMP levels [42,77]. It is unclear whether glucagon increases the cGMP levels in the human heart.

In the papillary muscle of dogs, the effects of isoprenaline and glucagon were additive, but those of Ca^2+^ and glucagon were not [32]. In other words, isoprenaline and glucagon apparently (like on the LTCC, see above) act via the same signal, namely cAMP, while Ca^2+^ and glucagon do not share a common action via cAMP. In isolated, perfused rat hearts, glucagon concentration- and time-dependently increased the cAMP levels, the activity of the cAMP-dependent protein kinase (PKA), and phosphorylase [162]. In that old paper, no significance was calculated. However, based on inspection, relevant changes in these parameters started at different glucagon concentrations. The most sensitive parameter was phosphorylase, which was already stimulated by 1 nM of glucagon, the lowest concentration studied [162]. The effects started 1 min after adding 100 nM of glucagon [162], the earliest time point studied. All these data are compatible with a cause-and-effect relationship between glucagon, cAMP-dependent protein kinase, and force in the rat heart. However, the effects were studied without a glucagon antagonist and/or GLP-1 receptor antagonists in the experiment. Hence, the underlying receptor is not clearly defined, as alluded to above.

The positive chronotropic effect of glucagon in rat right atrial preparations was reduced by inhibitors of PKA with 3 µM of H-89 (Figure 1), but not with 10 μM of ruthenium red, an inhibitor of sarcoplasmic reticular Ca^2+^-ATP(adenosine triphosphate)ase (SERCA) function (Figure 1, [70]). In contrast, inhibitors of the activity of protein kinase C or Ca^2+^ calmodulin-dependent kinase inhibitor did not reduce the potency of glucagon to raise the beating rate in isolated rat right atrial preparations [70]. Surprisingly, for a process thought to be mediated by cAMP, the addition of rolipram (1 μM), cilostamide (0.3 μM), or 3-isobutyl-1-methylxanthine (IBMX) 3 μM failed to increase the potency of glucagon to raise the beating rate in rat right atria [70]. This might be a species-dependent observation, because IBMX under these conditions also did not potentiate the positive chronotropic effect of isoprenaline. However, the authors speculated that compartments of cAMP or PDEs might underlie their observations [70].

On the other hand, region-specific differences play an undeniable role in the signal transduction for glucagon. In adult rat ventricular cardiomyocytes, glucagon increased the cAMP levels (total cytosolic levels and also subsarcolemmal levels significantly), and this increase in cAMP was about doubled by 100 μM of IBMX or 10 μM of rolipram [86]. Likewise, glucagon increased the current through the LTCC in adult rat ventricular cardiomyocytes. This current was increased further by additionally applied rolipram, indicating that PDE IV is relevant for the signal transduction of glucagon, at least in adult rat ventricles [86]. Recently, in cardiac cells, methods have been reported on how one can measure the compartments to which glucagon peptide 1 receptor can couple [163]. Such an approach will probably be helpful for the glucagon receptor [164]. At least in hepatocytes, glucagon activates gene transcription via endocytosis [165]. A similar pathway might also be active in cardiomyocytes. In the liver, glucagon can lead to the acetylation of nuclear proteins and, thus, the activation of nuclear proteins [166]. It remains to be elucidated whether glucagon can also acetylate and activate nuclear proteins in the heart. In the isolated, perfused rat heart, glucagon increased the taurine influx into the heart [167]. The role of taurine in the signal transduction of glucagon remains to be determined.

In principle, the glucagon receptor can also signal through Gq and phospholipase C (liver: [124,168], rat mesangial cells: [119,169], and HEK cells: [170]). This would lead to increased inositol-1,2,5 trisphosphate (IP3) and 1,2-diacylglycerol (DG). DG would activate protein kinase C. IP3 could release Ca^2+^ from stores in cardiomyocytes and thus increase the cytosolic Ca^2+^, increasing the force in the heart (Figure 1). Protein kinase C can phosphorylate and alter the function of many proteins in the heart, such as phospholamban or ERK1/2. It might be worthwhile to use kinase inhibitors to clarify the role of the cardiac glucagon receptors acting via phospholipase C.

In HL-1 cells, a tumor cell line showing some resemblance to cardiomyocytes, transfected with the glucagon receptor, glucagon (20 nM, 24 h) increased the PPARα protein translocation to the nucleus and, consequently, probably augmented the mRNA expression of the PPARα-target genes, such as carnitine palmitoyltransferase 1b, via the MAPK-pathway [18]. In hearts, glucagon (given at 8 ng/g every 8 h for 24 h to living mice) elevated the phosphorylation state of pyruvate dehydrogenase (leading to reduced activity and less glucose oxidation) [18]. In HL-1 cells transfected with the glucagon receptor, glucagon at 20 nM for 3 h further enhanced H_2_O_2_-stimulated apoptosis markers (cleaved caspase 3), suggesting that glucagon can induce apoptosis in the mammalian heart. Usually, the effects of glucagon that are mediated via cAMP generation involve cAMP-dependent protein kinase. However, at least in hepatocytes (and hypothetically in cardiomyocytes), the effects of increased cAMP are also mediated by the cAMP-binding exchange protein (EPAC) [171].

## 8. Inotropic and Chronotropic Effects of Glucagon in Animal Hearts

As already alluded to, glucagon can increase the force of heart contraction in many, but not all, tested species. Concentrations as low as 40 nM of glucagon could increase the force of contraction in isolated, perfused rat hearts [172]. However, these positive inotropic effects (an increase in left ventricular pressure has been reported) are accompanied by a positive chronotropic effect (an increase in the frequency of the heartbeat, [172]). Now, changes in the beating rate alone can alter the force of contraction: the well known “treppe” or “staircase” phenomenon [173]. Hence, a change in chronotropy could increase the force of contraction in an isolated, perfused heart, even if the drug that induced the increase in the beating rate did not act on any receptor in the ventricle (for instance, because this receptor is simply not expressed in the ventricle). This seems to be the case in the guinea pig heart (Table 1): in the sinus node cells of the guinea pig heart, glucagon can increase the depolarization rate and thus the beating rate. This increase in the beating rate also occurs in the perfused heart of the guinea pig and is almost certainly mediated by this staircase phenomenon (sometimes called frequency-induced inotropy): in isolated, paced guinea pig ventricular preparations, glucagon is practically devoid of any positive inotropic effect (Table 1). To complicate matters, the staircase phenomenon shows a species dependence. There can be a positive of a negative staircase phenomenon. In other words, increasing the beating rate of the left atrium can, depending on the species studied, lead to a positive or negative inotropic effect. For instance, in rat atrial preparations and mouse atrial preparations, an increase in the beating rate reduces the force of contraction, whereas in guinea pig and human atrial preparations, an increase in the beating rate increases the force of contraction [174,175]. One can exclude these staircase phenomena simply by electrically pacing the heart. Indeed, in electrically driven isolated, perfused rat hearts, 10 nM or glucagon increased the left intraventricular pressure [45,68], suggesting that the inotropic effect of glucagon, at least in rats, occurs independently of changes in the beating rate. Another way to get rid of the staircase phenomenon is to use isolated papillary muscles, which have to be paced in order to contract. Indeed, glucagon increased the force of contraction in paced rat papillary muscles [3,23,44].

In early work on paced canine papillary muscles, glucagon starting at 0.25 μM exerted a positive inotropic effect [33]. This effect was attenuated by Mn^2+^, suggesting an inhibition of the currents through the LTCC ([33], compare Section 9). In parallel, glucagon increased the transport rate of Ca^2+^ into canine cardiac microsomes [33], suggesting that glucagon via cAMP increases and the phosphorylation of phospholamban increases the pumping rate of SERCA (Figure 1). Interestingly, in anesthetized dogs, glucagon not only increased the force of contraction, but also increased the coronary blood flow, suggesting a dilation of the coronary arteries by glucagon [33]. This better perfusion of the heart might also contribute, albeit indirectly, to a positive inotropic effect of glucagon. As mentioned above, when we were discussing the expression of the glucagon receptor in the whole heart, one would expect to detect mRNA of the glucagon receptor in mRNA isolated from whole hearts, simply because one would also measure the expression in mRNA derived from cardiac smooth muscle cells [44]. In rat ventricles, there is a positive inotropic effect (PIE) of glucagon potentiated by IBMX. Glucagon protects the isolated, perfused cavian hearts against anaphylactic responses [176].

As already mentioned, the potency of glucagon to increase the force of contraction in muscle strips from rats’ right ventricles was increased by cilostamide (a PDE 3 inhibitor, [85]). Interestingly, cilostamide under these conditions did not potentiate the effects of dobutamine, a β-adrenoceptor. Hence, the role of PDE3 for cAMP generated by the glucagon receptor and β-adrenoceptor stimulation must differ in rat ventricles [85]. In other words, compartments for glucagon-stimulated cAMP molecules might exist. Interestingly, in isolated, perfused hearts, the positive inotropic effect of glucagon was attenuated by the application of methoxamine, an alpha1-adrenoceptor agonist. It has been suggested that methoxamine might decrease the cAMP elevated by glucagon, because methoxamine either stimulates PDE activity or inhibits the activity of AC via Gi [177].

The inotropic effects of glucagon in the animal heart seem to be mediated by the LTCC: on potassium-depolarized cardiac preparations, glucagon increases the force of contraction [178]. This is typical for agonists acting also via the LTCC (see Section 9 for details). Glucagon can induce a positive chronotropic effect in the mouse heart. This effect is likely not indirectly mediated by the GLP1-1 receptor in the sinus node of the mouse, because the GLP-1-receptor agonist liraglitide (up to 0.3 μM) failed to increase the beating rate in isolated right atrial preparations from mice, but was able to increase the heart rate when injected into living mice [19].

In summary, the positive inotropic effect of glucagon shows a species dependency (see Section 10): for instance, there is no inotropic effect of glucagon in rabbits. Moreover, the positive inotropic effect of glucagon can be region specific (see Section 11): in rats, there is no positive inotropic effect of glucagon in the left atrium, but there is a positive inotropic effect in the guinea pig ventricle. Such species and regional differences in mammalian hearts are not without precedence, but have been described before for other receptors that stimulate cardiac AC. Examples would be H2-histamine receptors or 5-HT4-serotonin receptors [179,180].

## 9. Electrophysiological Effects of Glucagon in Mammalian Hearts

In pancreatic cells, glucagon can reduce the ATP-sensitive current I(K.ATP), as shown in mouse pancreatic cells via a Ca^2+^/calmodulin-dependent pathway [181], while GLP-1 does not exert effects on I(K.ATP) in these cells, but slows the inactivation of the calcium current (I(Ca)) [182]. Since both currents are also present in cardiomyocytes, one could imagine that glucagon and GLP-1 may also have effects on cardiac electrophysiology.

Surprisingly, in guinea pig right ventricular preparations, glucagon prolonged the duration of the monophasic action potential and reduced the upstroke velocity, which were shortened or unaltered by adrenaline in this model, respectively [183]. The authors argued that these alterations were similar to the actions of the antiarrhythmic drug procainamide, and thus glucagon might be regarded as an antiarrhythmic drug ([183], see also below). However, in anaesthetized dogs, a direct coronary bolus injection of glucagon not only induced tachycardia, but also transient arteriovenous nodal rhythms [30]. In dogs, when the His- bundle has been surgically destroyed and a spontaneous secondary pacemaker in the ventricle occurs, the beating rate could be elevated by glucagon [184]. Intraventricular conduction in patients was enhanced by glucagon [185]. In rat and frog cardiomyocytes, glucagon increased the current through the L-type calcium channel (LTCC). However, in frog cardiomyocytes, the increase in the LTCC was mediated by the inhibition of PDEs, whereas in rat ventricular cardiomyocytes, the increase by glucagon in the LTCC was mediated by glucagon stimulating AC, thus increasing the cAMP levels and activation of PKA [42,77]. Moreover, equal concentrations of glucagon were more effective at increasing the current through the LTCC than in frog cardiomyocytes. This is regarded as evidence that different cAMP pools may exist in rat compared to frog cardiomyocytes. In rats, the effects of isoprenaline and glucagon on the increase in the current through the L-type calcium channel (LTCC) are not additive, suggesting that both drugs finally use the same pathways, namely the stimulation of AC, to increase the force of contraction [42]. However, further investigations have revealed that the glucagon effect is sensitive to PDE IV inhibition, so the authors concluded that the specific functional coupling of individual PDE families to certain Gs-coupled receptors act as a major mechanism enabling cardiac cells to generate heterogeneous cAMP signals in response to different hormones [86].

In isolated cardiomyocytes from adult canine left ventricles, it was shown that GLP-1 at 5 nM increased I(Ca) by 23 ± 8% and markedly prolonged the durations of the action potentials by 128 ± 36 ms (*p* < 0.01) and 199 ± 76 ms (*p* < 0.05) at 50% and 90% repolarization, respectively [186]. N’1-(3,3,6,8-tetramethyl-1-oxo-1,2,3,4-tetrahydronaphthalen-2-yliden)-2-cyanoethanohydrazide (TTYC), a synthetic drug that increases the secretion of glucagon-like peptide-1 and the intracellular Ca(^2+^) concentration in GLUTag cells, was shown to enhance I(Ca.L) in isolated rabbit ventricular myocytes and exert positive cardiac inotropic effects in vivo in anesthetized rats. The effect on the I(Ca.L) was described as an enhancement of the peak current, a left shift (a shift to more negative potentials) of steady state activation by 15 mV, and a slowing of the inactivation of the calcium current [187].

As far as we could find out, the effect of glucagon on the LTCC has not been directly measured in atrial or ventricular human cardiomyocytes using any electrophysiological technique, and this may be another study needed. There is, as mentioned elsewhere, only evidence that the effect of verapamil, an LTCC antagonist, on the force of contraction is antagonized in the human heart. Hence, it is likely that glucagon can increase the current through the LTCC in the human heart, but this is very indirect evidence. In principle, glucagon receptor stimulation (4 nM of glucagon) does not need to stimulate ion channels via the cAMP and PKA systems. At least in primary rat hepatocytes, increases by glucagon in ion currents were not blocked by H89 (a PKA inhibitor), but by Epac inhibitors [188]. Moreover, in that study, phospholipase C was also shown (using U73122) to increase cell membrane ion currents. Whether the same holds true in cardiomyocytes remains to be assessed [188].

Another missing aspect is that, to the best of our knowledge, the effect of glucagon and GLP-1 on cardiac I(K.ATP) has not been evaluated. In addition, although chronotropic effects can be explained by the effects on I(Ca.L), it is known that drugs acting on chronotropy involving I(Ca.L) may also exert effects on the funny current I(f) [189], an aspect that has not been investigated for glucagon or GLP-1 so far.

## 10. Species

Glucagon elevates, in the isolated atria of dogs, cats, and guinea pigs, but not in rabbits, the force of contraction [3]. Consistent with the lack of a glucagon-induced positive inotropic effect in the rabbit heart, glucagon did not increase the AC activity in preparations from rabbit hearts [66]. Species differences occur, which makes it challenging to translate animal data to the situation in patients directly. In rat cardiomyocytes, an agonist at glucagon-like peptide-1 receptors increased the cAMP levels, but reduced the contractility. This was accompanied by a decrease in the pH in the cardiomyocytes, and was interpreted as the subsequent desensitization of the myofilaments to calcium ions [118].

Regarding cAMP levels, interesting species differences have been recorded. In paced isolated, perfused rat hearts, 0.1 μM of glucagon induced a time-dependent increase in the cAMP content that preceded a PIE [45]. In contrast, in isolated, perfused, and paced guinea pig ventricles, 0.1 µM of glucagon failed to increase the force or cAMP levels [45,59]. However, there is the problem that, while glucagon did not stimulate AC in the guinea pig ventricle, glucagon was able to inhibit the activity of the milrinone-inhibitable PDE activity [77]. Hence, if this inhibitory action of glucagon on PDE III occurred in the cardiomyocytes of the guinea pig, then why was the force of contraction not increased? A similar question can be raised in the mouse ventricle. In the adult mouse ventricle, glucagon did not increase the force of contraction [18]. There can be some criticism of this report. Looking closely at the original recording, one detects at least intermittent ventricular fibrillation in these perfused mouse hearts. Hence, one would encourage further studies on mouse hearts without any arrhythmias, adding to them glucagon to address this question more convincingly. In the adult mouse ventricle, glucagon can inhibit PDE III [77]. It is possible that this inhibition of PDE III is not functionally relevant, because it depends on the homogenization of tissue, which might have broken the physiological signal transduction pathways and caused artifactual signal transduction. Furthermore, PDE III inhibition might have increased the cAMP in a cardiomyocyte compartment not coupled with the force of contraction.

Stimulation of the current through the LTCC was antagonized by acetylcholine in rat ventricular cardiomyocytes, but not in frog ventricular cardiomyocytes, which is consistent with the species-dependent alternative modes of signal transduction [42]. In membrane preparations from frog hearts, glucagon inhibited the PDE III activity, but not in membrane preparations from rat hearts [42,77]. However, this is organ specific; in rat hepatocytes, glucagon stimulated AC and inhibited the PDE IV activity [190]. This inhibitory action of glucagon on rat liver PDE IV activity was pertussis toxin sensitive [190]. Moreover, glucagon was much more potent in increasing the current through the LTCC (half maximum effect: EC_50_ = 41 nM) in frog ventricular cardiomyocytes than in rat ventricular cardiomyocytes (EC_50_ = about 400 nM, [42]). Glucagon was 15 times less effective at increasing the current through the LTCC than isoprenaline in frog ventricular cardiomyocytes [42]. This suggests that PDE inhibition is not a powerful way of increasing cAMP, at least in rat ventricular cardiomyocytes [42]. The inhibitory effect of glucagon in the frog heart was gone when membranes were incubated with pertussis toxin, suggesting signal transduction via inhibitory GTP-binding proteins [77].

It has been suggested that, in species where glucagon does not activate cardiac ventricular AC, such as monkeys, mice, and guinea pigs, glucagon might increase the force of contraction by inhibiting the PDE activity [42]. This has been confirmed, at least in mouse and guinea pig ventricles (Table 1). In particulate preparations from the mouse ventricle or guinea pig ventricle, glucagon (reaching a plateau at 1 µM of glucagon) inhibited the PDE activity [77]. One would predict that, in frog and guinea pig cardiac preparations, giving a submaximal concentration of a PDE inhibitor should reduce the potency of glucagon to increase the force. Moreover, if whole guinea pigs were injected with pertussis toxin, one would expect that glucagon would not increase the force of contraction in isolated ventricular preparations.

However, in rabbit livers, but not rat livers, glucagon led in vivo to an increased phosphorylation state of the so-called inhibitor 1 of protein phosphatase 1 [191]. This latter finding indicates that the species specificity of glucagon action is a general phenomenon and not an exceptional finding in the heart. Moreover, because we have shown that isoprenaline increases the phosphorylation state of inhibitor 1 in the isolated, perfused guinea pig heart [192], whether this protein is stimulated by glucagon in the mammalian heart in general and the human heart in particular remains to be studied. This would add an additional layer of crosstalk in protein phosphorylation due to the glucagon in the heart, because the phosphorylation of inhibitor 1 augments the phosphorylation of regulatory proteins such as phospholamban.

## 11. Region

No contractile effect of glucagon has been noted in the electrically stimulated left rat atrium or electrically stimulated rat right atrium (sinus node had been removed, [67]). Furthermore, no inotropic effect was apparent in the atrium in the presence of IBMX, although glucagon receptors were detected with Western blot [67]. The authors hypothesized that glucagon might have been coupled in the rat atrium with Gs and Gi. Hence, any stimulatory effect on Gs might have been compensated for by the stimulation of Gi. Hence, they treated rats with pertussis toxin and functionally inactivated Gi [67]. Nevertheless, glucagon could not raise the force in the atrial preparations from the hearts of these pertussis-toxin-treated rats [67]. It was reported that, using other antibodies in Western blots, the expression of the glucagon receptor in rat ventricles was several times higher than that in the rat atrium [67].

These results may explain, in part, the functional differences in the rat heart [67]: in the rat ventricle, glucagon increases the force of contraction, but not in the left atrium of the rat [67]. However, glucagon increases the beating rate of isolated rat right atrial preparations [70]. The density of the mRNA of the glucagon receptor is about three times higher in rat sinus node cells compared to the rest of the atrial rat muscle tissue [70]. This does not necessarily predict the protein level of the glucagon receptor in the rat heart. However, the authors argue that the functional effects of glucagon in the rat atrium heart might follow mRNA receptor densities. Thus, glucagon fails to raise the force of contraction in the rat’s right atrium (see below and Table 1), but induces positive chronotropic effects, simply due to more receptors being stimulated in the sinus node because there are more glucagon receptors [70].

In contrast, glucagon (1 µM) increased the beating rate in guinea pig right atrial preparations. However, it did not increase the force of contraction in paced ventricular guinea pig preparations [45]. The regional expression of the mRNA of the glucagon receptor in the mouse heart exhibits the following rank order: by far, the highest expression is in the right atrium, then the left ventricle, left atrium, and a faint band in the right ventricle (single experiment: [18]). Protein data were not reported [18]. GLP1-R mRNA was highest in the mouse right atrium, then much less in the mouse left atrium, and was missing in both the mouse left ventricle and mouse right ventricle [18]. GLP2-receptor mRNA was missing in mouse hearts, but was present in mouse livers used as a positive control [18].

## 12. Inotropic and Chronotropic Effects of Glucagon in Isolated Human Hearts

A decisive question, in our view, is whether glucagon increases the force of contraction in the human heart. In addition, the receptor that is stimulated by glucagon in the human heart must be very clear before any cardiac drug therapy with glucagon in humans makes sense. In the human ventricle, glucagon alters the electrophysiological parameters [61], but not the force. Likewise, in human atrial preparations, glucagon has been claimed to be ineffective, but supporting data were not shown [62].

We assembled the data available from studies on glucagon in the human heart in Table 1. Significantly, from the early days, the inotropic effects of glucagon in isolated human heart samples have been recorded. With the first cardiac surgery in the 1950s, this method became available. Some studies have failed to detect a positive inotropic effect in the human heart, either in human atrial or ventricular preparations (Table 1). At present, one can only speculate why some investigators have failed to detect the PIEs of glucagon. Methodological differences (buffer composition) or, more likely, co-medication or comorbidities of patients come to mind. Nevertheless, more efforts are encouraged to resolve this obvious discrepancy in the literature.

Another problem in previous studies is the potency of glucagon. Contractile studies need much higher glucagon concentrations (1 µM and more). This is much higher than the physiological concentration (5 pM, see above), but also higher than the EC_50_-value of glucagon to stimulate AC in transfected human glucagon receptors (around 10 nM). Hence, if the glucagon receptor mediates the cardiac effects of glucagon, why is the glucagon receptor so insensitive to glucagon in human cardiac tissue? Is there a different signal transduction mechanism used besides cAMP? It is unlikely, but not impossible, that glucagon is rapidly degraded in human muscle strips. This could be studied using sensitive mass spectrometry methods. Then, much higher glucagon concentrations would be required to stimulate a human cardiac glucagon receptor. Another explanation would be that glucagon does not act via the stimulation of a high-affinity glucagon receptor in the human heart, but uses another low-affinity receptor. This could be the glucagon-like protein 1 receptor with an affinity for glucagon and an EC_50_-value of about 100 nM.

Therefore, experiments should be performed using antagonists for glucagon receptors and antagonists at the GLP-1-R. The prediction would be that only GLP-1-R antagonists, but not glucagon receptor antagonists, would antagonize the inotropic effects of glucagon in human cardiac tissue.

Additional research could study the contractile effects of glucagon-isolated human atrial and ventricular cardiomyocytes. This is important because it is conceivable that glucagon acts in intact human cardiac tissue on non-cardiomyocytes (e.g., neural axons) and releases mediators that increase the force of contraction. To this end, one could isolate human cardiomyocytes with collagenase to obtain primary cultures. Alternatively, induced stem cells could be used to obtain cardiomyocytes with atrial or ventricular gene expression profiles. Such cells typically beat spontaneously and are thus an excellent model for assessing glucagon’s inotropic and chronotropic effects. With some limitations, transgenic mice could be used in this regard. One could use mice with a knockdown in the human glucagon receptor or human GLP-1-R in mice (generally or heart specific), and then study which glucagon concentrations are needed to increase the force of contraction or beating rate in these transgenic mice compared to wild-type mice.

The situation is different in vivo in humans. In our view, the literature is robust. Parenterally used glucagon in a sufficiently high dosage could have a positive chronotropic effect. Using the positive force–frequency relationship in the non-failing human heart (“staircase” or “Treppe” phenomenon, [173,175]) will physiologically exert a positive inotropic effect. Conceivably, a direct positive inotropic effect occurs, regardless of this positive chronotropic effect. In addition, these studies have obvious limitations. It might be that the positive chronotropic effects are, at least in part, only indirect. Glucagon is known to probably reduce peripheral arterial resistance via the glucagon receptor and cAMP (Table 1 for exemplary studies). In a reflex mechanism, the human heartbeat rate would increase to compensate for this reduced resistance by increasing the cardiac index. This might partly occur due to an adrenergically induced increase in the beating rate. These mechanisms could be addressed in further clinical studies with glucagon receptor antagonists or β-adrenergic antagonistic drugs.

## 13. Clinical Relevance

### 13.1. Glucagon in Heart Failure

The plasma concentrations of glucagon are at least 10 times lower than the glucagon concentrations that increase the force of contraction under certain conditions [42]. For example, in human peripheral venous blood, arterial blood, and portal venous blood, low glucagon concentrations of 23 pico mole per liter (pM), 20 pM, and 48 pM have been reported (review: [93]). However, in disease, the role of glucagon may change and does not rule out the role of glucagon as a drug for increasing the force of contraction. As discussed above, at least one study on heart failure patients exhibited 700 pM of glucagon [143]. Moreover, there are glucagon-producing tumors in patients called glucagonomas that have been accompanied by and might have caused heart failure [193,194,195,196,197]. A causal relationship is sometimes made likely when the surgical removal of the glucagon-producing tumor is accompanied by an improvement in or disappearance of an accompanying heart failure, as was reported, for example, by octreotide therapy [197] or surgical removal of the tumor [194].

There are old clinical data that describe clinical improvement when glucagon infusion is added to the usual therapy of chronic and acute heart failure [198]. Glucagon has been successfully applied in chronic heart failure patients [64]. Clinically, glucagon use is accompanied by nausea and vomiting in patients during clinical heart failure trials [199]. This nausea is probably due to glucagon receptor stimulation in the area postrema of the brain [200]. However, other studies on the mechanism(s) of this glucagon-induced nausea have concluded that peripheral mechanisms, such as an alteration in intestinal uptake of ions after a glucagon-induced relaxation of the smooth muscles of the intestine, are more important [201].

If the evidence is weighed, the role of glucagon in the heart itself seems to depend on whether a non-failing heart or heart with acute or chronic failure is studied (Table 1). Moreover, whether any beneficial effect is derived from action on cardiomyocytes, the sinus node, or the vasculature is unclear. The coronary arteries might dilate (rat aorta, [202]), or peripheral resistance probably declines after glucagon [63]. Whether these changes lead to a better patient prognosis is only supported by small, open-label studies, and is thus unclear.

In hypertension-induced cardiac hypertrophy in rats, the efficacy of glucagon in raising the left ventricular pressure was less than that in control rat hearts, whereas the positive chronotropic effects of glucagon remained unaltered [203]. In cardiac failure with a low systolic blood pressure, glucagon was tried not as a single bolus, but as a 5 mg per hour intravenous infusion for several days. This led to an increased systolic blood pressure and clinical improvement in patients [204]. However, in about 10% of patients, side effects, often nausea, forced a termination of the study [204]. Similarly, glucagon also acts as an agonist at the GLP-1 receptors and can lead to nausea. In this case, it was suggested that nausea reduced interest in eating, leading to weight loss, and is a helpful side effect of such drugs [205]. Hence, one should try to find a way to stimulate the cardiac glucagon receptor, but not the glucagon receptor in the area postrema. Theoretically, a biased agonist might stimulate the cardiac glucagon receptors, but not the nausea-inducing central glucagon receptors. However, much more work is required to find such a biased agonist at the glucagon receptor.

### 13.2. Glucagon in Cardiac Ischemia

Low glucagon concentrations, such as 8.7 nM, did not increase the force in isolated, perfused rat hearts, but protected isolated, perfused rats, to some extent, against hypoxic damage [206]. In more detail, these authors perfused a group of isolated rat hearts with an oxygenated perfusion buffer and compared this with a buffer that was lacking oxygen [206]. Therefore, they measured under hypoxic and not ischemic conditions. Ischemia would have been achieved if they had stopped the pump that perfused the heart with the buffer. Their read out was the force generated in the apex of the heart. Moreover, they electrically stimulated the hearts slightly above the intrinsic beating rate [206].

In a mouse model of myocardial infarction, an antibody against glucagon improved cardiac function, and one might speculate that such an approach might be feasible in humans [207]. In support of this concept, it was reported that mice with a deletion of the glucagon receptor, in general or selectively in the heart, fared better after myocardial infarction [18,208]. Now, one might compare the apparently contradictory findings in rat and mouse hearts: in the rat heart, glucagon is protective [18]. However, glucagon appears to be detrimental in mouse hearts, because an antibody that presumably stops any signal transduction through the glucagon receptor is beneficial [18]. Several explanations might account for this obvious discrepancy: it might be simply species differences between mouse and rat hearts. There could, alternatively, be just methodological differences: the rats were electrically stimulated and this might have offered some level of protection for the rat heart. Moreover, in the rat hearts, hypoxia was achieved, whereas in the mouse hearts, global no-flow ischemia was achieved. It is conceivable that, in the mouse heart, other biochemical pathways to those in the rat heart may have been involved. Global ischemia in mouse hearts would be expected to lead to much more damage than continued, albeit hypoxic, perfusion. As one example, in hypoxia and ischemia in the heart, the production of detrimental free radicals is increased. These free radicals would be washed out continuously in hypoxic but perfused rat hearts. These radicals would stay in mouse hearts under global ischemia, and high concentrations of detrimental free radicals would build up. We could speculate that the situation in the globally ischemic heart might be clinically more similar to a cardiac transplantation and would predict that glucagon is deleterious in this situation. Blocking glucagon receptors with drugs or antibodies, might increase the success rate of transplantations [18].

### 13.3. Glucagon to Treat Intoxication

In early work on living dogs, propranolol was used to decrease the rate of the depolarization of the sinus node or, using a direct injection in the atrioventricular (AV) artery, the conduction rate through the AV node [209]. These effects of propranolol, which might occur in intoxication, were eliminated using glucagon in dogs [209]. This finding is consistent with the assumption that propranolol intoxication can be treated with intravenous glucagon. In isolated, perfused rat hearts, glucagon could increase the force of contraction previously reduced by verapamil. This was interpreted to support the clinical use of glucagon to treat intoxications due to Ca^2+^ antagonists [210].

Clinically, glucagon has been suggested to help increase the force of contraction in patients in whom contractility was impaired by the simultaneous administration of L-type calcium ion antagonists or β-adrenoceptor antagonists [4,211]. Intravenous glucagon helped to treat intoxication by β-adrenoceptor antagonist intoxication [198]. A high dosage of intravenous glucagon has been successfully used to treat tricyclic antidepressant intoxication [212,213]. Glucagon is still used to treat intoxication with β-adrenoceptor antagonists [214]. However, the efficacy of this treatment is not fully understood [214], and these concepts have been questioned. It has been argued that, in some clinical studies, no apparent positive inotropic effect of glucagon at the recommended doses could be observed. A placebo effect might be involved, or glucagon might dilate the peripheral arterial vessels, leading to reflective tachycardia and a secondary increase in contractility [61]. In an older study, glucagon was able to antagonize the bradycardic effects of propranolol [215]. In a recent study with healthy volunteers, high-dose glucagon could antagonize the cardiovascular effects of esmolol (a β-adrenoceptor antagonist), suggesting that the current guidelines are at least reasonable [124].

### 13.4. Glucagon and the Diabetic Heart

Some have claimed that glucagon might contribute, at least in part, to the development of type 2 diabetes (review: [216]). Type 2 diabetes impairs cardiac function by increasing the incidence of heart failure, notably heart failure with a preserved ejection fraction. The latter is of growing incidence in patients. Hence, there is currently considerable interest in companies and academia to devise drugs that block the glucagon receptors, thus preserving cardiac function longer as we age. An inactivating antibody at the glucagon receptor reduced the incidence of diabetic cardiomyopathy in a mouse model of diabetes when given over several months [217]. There has been some success in devising drugs comprising glucagon, a linker, and triiodothyronine (T3). This bifunctional agonist was helpful in a mouse model of diabetes for preserving cardiovascular function [123].

### 13.5. Glucagon and Arrhythmias

There have been reports of an antiarrhythmic effect and proarrhythmic effect of glucagon in the mammalian heart. Because glucagon has been convincingly demonstrated to increase cAMP, it is expected, based on experience with β-adrenergic drugs, that glucagon would be arrhythmogenic. However, it is possible that some additional mechanisms of action of glucagon, besides the stimulation of cAMP, such as the action of poorly understood ion channels, play a beneficial role. In addition, the prevalent disease type in a patient’s heart might play a role.

Moreover, there is the question of how to define an arrhythmia. In patients, tachycardia is defined as a beating rate of more than 100 beats per minute, and this considered to be arrhythmia. In this broader sense, glucagon is clearly arrhythmogenic (see Table 1). An early study involved anesthetized (morphine and pentobarbital), closed-chest dogs, in which glucagon was infused into the left coronary artery (50–70 µg, [180]). This bolus of glucagon led to transient tachycardia and a transient arteriovenous nodal rhythm [30]. In dogs, glucagon sped up AV conduction in situ. However, in contrast to β-adrenergic catecholamines, glucagon did not increase the ventricular automaticity in canine hearts [218]. However, using a different protocol (pacing the sinus node), others have noted that glucagon could increase the ventricular automaticity in dog hearts [219].

Likewise, others have reported that glucagon could increase the ventricular automaticity in dogs [184] or shorten the duration of the action potential in spontaneously beating canine Purkinje fibers [34,220]. The direct infusion of glucagon into the AV-nodal artery in dogs increased the conduction through the AV node [209]. Some have reported that glucagon might be antiarrhythmic in myocardial infarction, at least in dogs [221,222]. In dogs, glucagon effectively treated ouabain-induced cardiac arrhythmias [222,223]. The guidelines recommend intravenous glucagon in patients with bradycardia as one treatment option [224]. During cardiac defibrillation, in order to treat ventricular fibrillation, after successful termination in a number of patients, the heartbeat did not restart. In a canine model of this complication, glucagon could significantly restore pacemaker activity [225].

In contrast, at least one report concluded that, even in healthy volunteers, glucagon infusion could induce arrhythmias [226]. Similarly, when glucagon was applied to facilitative enteroscopy (by the inhibition of gut wall movement), paroxysmal supraventricular arrhythmias were reported [227]. Likewise, in pigs, when the anterior descending coronary artery was ligated, high local concentrations of glucagon-induced arrhythmias were observed [228].

There are data suggesting that 2–6 mg of glucagon, intravenously, can be beneficial in AV-block type 1 and type 2 (Wenckebach-type and Mobitz-type) and AV-block type 3 in adult patients [229]. In children with third-degree AV-block, intravenous (but not intramuscular) glucagon could transiently increase ventricular beating [230]. The author found 0.05 mg/kg of glucagon to be equieffective and longer lasting than isoprenaline [230], and noted that he saw arrhythmias with isoprenaline, but not with glucagon, in these treated children [230]. However, the study is old and the number of patients was low. Hence, novel studies using up-to-date methodology on this issue are awaited with interest.

With regard to GLP-1, in a large meta-analysis comprising >70,000 patients, GLP-1 agonists were not found to principally increase the risk of arrhythmia in diabetic patients. Only at high doses in patients with a high body mass index (BMI) was there a risk for ventricular arrhythmias [231]. Similarly, dulaglutide was not associated with an increased risk for cardiac arrhythmia in a randomized clinical study [232]. The problem with such findings is the rather complex pathophysiological situation comprising glucose control, energy metabolism, body mass index, other risk factors such as blood pressure, and metabolic disease, etc., so it is not possible to conclude a direct antiarrhythmic effect from these observations.

Exendin-4, a GLP-1 agonist, was investigated in anesthetized male rats pretreated with exendin-4 (5 μg/kg, intravenously) 1 h before ischemia in the absence and/or presence of 5-hydroxydecanoic acid (5-HD, 10 mg/kg, intravenously, a specific inhibitor of mitochondrial ATP-sensitive potassium (KATP) channels, given 10 min before ischemia). Thereafter, the rats were subjected to ischemia for 30 min. The duration of ventricular tachycardia (VT) and ventricular fibrillation (VF) and the sum of the VT and VF episodes were significantly reduced by exendin-4. This antiarrhythmic effect was completely absent if the animals were pretreated with 5-HD, indicating a sensitivity of the exendin-4 effect to the K.ATP channels [233]. Thus, this may be a hint that further investigation of the effects of glucagon and GLP-1 on mitochondrial and sarcolemmal I(K.ATP) is needed.

### 13.6. Glucagon Receptor Mutations and Functions

Loss of function mutations in humans exist for the glucagon receptor [234]. For instance, a point mutation of the glucagon receptor reduces the affinity for glucagon. This mutation may contribute to diabetes mellitus II [235] and hypertension [236]. If this mutation is also present in the human heart, glucagon should be less effective at raising the beating rate or force of contraction. Practically, this would mean that glucagon is not indicated in patients harboring a loss of function mutation of the glucagon receptor to treat an intoxication with a negative inotropic compound such as verapamil or propranolol. A possible experimental test for this prediction would be to exactly overexpress in mouse hearts such a mutated glucagon receptor and evaluate its cardiac function in vitro and in vivo. Missense glucagon receptors probably also couple less to arrestins [237]. This may contribute to the detrimental cardiac effects of such mutations.

## 14. Glucagon Outside the Heart

Sodium-glucose transporter-2-inhibitors (SGLT2 inhibitors) have gained acceptance in nephrology and heart failure treatment. An added benefit is that SGLT2 inhibitors seldom induce hypoglycemia, but usually increase satiety, thus leading to weight loss [238]. As it turns out, in clinical studies, SGLT2 inhibitors can increase the glucagon levels in the blood [239]. Some have argued that this is a direct effect, as SGLT2 inhibitors are expressed in te α-cells in the pancreas. Others have argued that the “glucagonotropic” effects of SGLT2 inhibitors are indirect; SGLT2 inhibitors reduce the glucose levels in the renal system [240]. No direct effect of SGLT2 activity on glucagon secretion has been noted. However, this is dependent model in db/db mice; an SGLT2 inhibitor (dapagliflozin) reduced, at the protein level, the expression of the glucagon receptors in the liver of treated animals [241]. It would be interesting to study the glucagon receptor expression in these mice or the hearts of diabetic patients. In some diabetic patients, the glucagon blood levels are elevated. This has led to the idea of treating patients with GLP-1 receptor agonists. This treatment should lead to reduced glucagon release in the pancreas and should thus normalize the blood glucose levels.

Moreover, there are some studies in which glucagon antagonists were given to patients with type 2 diabetes [242]. Regrettably, side effects on liver function (steatosis) or increases in blood cholesterol led to the cessation of these investigational drugs [243]. There are ongoing trials with glucagon receptor agonists alone or in combination with GLP1-agonists, as well as with drugs that are bifunctional; that is, they stimulate glucagon receptors and GLP-1-receptors with one molecule. The initial results of such trials indicate that such dual-receptor stimulation promotes mitochondria function in the liver, reduces glucose blood levels, and lowers the body weight in obese patients e.g., [244,245].

## 15. Summary

Currently, a few but critical clinical indications for glucagon are commonly accepted. For example, self-injections of glucagon are indicated to treat hypoglycemia. This hypoglycemia might seldom occur after bariatric surgery, more often as post-exercise-induced hypoglycemia. Another experimental indication is pumping glucagon parenterally and insulin to treat diabetes. This mixture is intended to prevent insulin-induced severe hypoglycemia. Another seeks to treat diabetic patients with orally available glucagon receptor antagonists, with some success [113]. Glucagon can increase the beating rate and force of contraction, at least in animal hearts. The level of glucagon and, thus, its cardiovascular action, can be altered in diseased states. Most studies agree that glucagon affects the beating rate and force of contraction in the human heart. However, there are conflicting data on whether the cardiac effects of glucagon in humans show regional differences.

It is conceivable that drugs that reduce the formation or degradation of glucagon might alter cardiac function. Research is needed to determine whether glucagon directly or indirectly can induce arrhythmias or whether it is an antiarrhythmic drug. Moreover, an exciting line of research is to determine whether cardiac glucagon receptors are targets for drugs against ischemia and reperfusion injury. In addition, there is a hypothesis that glucagon receptor antagonists might gain importance in treating insulin-independent diabetes mellitus. Finally, little is known about how precursor proteins or glucagon degradation products might allosterically alter the receptor-mediated function of glucagon and whether they should become drugs or drug targets.

## 16. Conclusions and Future Directions

Despite about 100 years of research, the cardiac role of glucagon in the human heart in health or disease is still open to serious questions and conflicting findings. From the present review, it seems clear that species differences and regional differences in the contractile response to glucagon in experimental animals (Table 1) are most likely due to higher or lower (below detection limits) expression levels of cardiac glucagon receptors and/or differences in the signal transduction pathways employed (Table 2, Figure 1). Clinically demanding is the question of why, in human isolated heart preparations, some groups find a positive inotropic effect of glucagon and others fail to do so (Table 1). It seems unlikely, but could be tested with PCR, whether these expression differences of glucagon receptors in the atrium and ventricles of the human heart exist due to genetic differences from country to country or even clinical center to clinical center. Alternatively, and in our view, more likely to explain these differences are the underlying diseases of the patients entering these studies. One could speculate that even different drug treatments used in different clinical centers might alter the expression of the glucagon receptors and therefore the cardiac action of glucagon in patients. Newer contractile studies with glucagon and in the absence and presence of glucagon receptor antagonists are required to close this knowledge gap. Moreover, further prospective clinical studies are deemed necessary to assess the cause-and-effect relationship between heart failure and even heart failure subtypes (with reduced or preserved ejection fraction), plasma glucagon levels, cardiac glucagon receptor densities, and contractile response in vivo and vitro to glucagon.

## Figures and Tables

**Table 1:** Species- and age-dependency of glucagon-induced increases in cardiac contractility in mammals, including humans.

	Left Atrium	Right Atrium	Ventricle	Remarks
Cat		[3,5,25] PCE [25] PIE	[5,25,26] In vivo: PIE [5,27] PIE: papillary muscle [27] PIE: perfused heart [28] No PIE in heart failure [25,29] PIE in heart failure	
Dog	[3,4] PIE	[3,4,30,31,32,33,34,35,36,37,38,39,40] PCE [31,36] PIE	[3,4,34,35,39,40] PIE [4] failing dog ventricle	[40] Coronary perfusion enhanced
Embryon-ic chick heart			[41] PCE	
Frog			[42] PIE	
Guinea pig	[3,43,44,45,46,47,48,49,50,51,52,53,54,55,56,57] PIE	[43,44,45,47,58,59] PCE	[44,45] No inotropic effect	
Human cardiac tissue (isolated)	[50] No inotropic effect	[23,50] No inotropic effect	[50,60,61] No inotropic effect, [60] APD shortened, in vivo: PIE [49,62] PIE in left ventricular papillary muscle strips from failing hearts	
Human patient or healthy volunteer In vivo	[38,51,52,53,63,64] PCE		[38,51,52,53,63,64] PIE (1–5 mg i.v.)	[38,52,63,64] Vascular peripheral resistance decreased, [38,51,52,63] nausea [51,63] vomiting, [64] flushing, [64] palpitations, [64] diarrhoea, and [64] hyperglycemia [52] Coronary flow increased, [52] oxygen consumption increased, [52] Blood glucose increased
Monkey			[42] PIE	
Mouse adult		[18] PCE, [54] No PCE	[50] No PIE	
Mouse fetal and neo-natal		[65] PCE late-term fetal mouse heart	Neonatal mouse cardiomyocyte [54,65] PIE, [55,56,57] PCE	
Rabbit		[66] No PCE	[3,66] No effect	
Rat, adult	[3] No inotropic effect [67,68] PIE	[23,44,67,68,69,70] PCE [3] No inotropic effect	[3,44,45,68,70,71] PIE	[44,68] Relaxation shortened
Rat, fetal		[54,72] No PCE		

PCE: positive chronotropic effect, PIE: positive inotropic effect, APD: action potential duration, and i.v.: intravenously.

**Table 2:** Species- and age-dependency of signal transduction pathways used by glucagon in cardiac preparations from mammals, including humans.

Species	Right Atrium	Left Atrium	Ventricle	
Cat	[28] AC stimulation in normal heart [28] No stimulation of AC in failing hearts		[74] AC stimulation [74] PDE not inhibited	
Chicken embryonic ventricular cardio-myocytes	[75] Calcium transients increased			
Dog	[31] AC not stimulated	[31] AC not stimulated	[76] cAMP increase [31] AC stimulated	
Embryonic chick heart	[41] AC stimulated		[41] Glucagon binding increased with age	
Frog heart	[77] PDE inhibition	[77] LTCC stimulation		
Guinea pig ventricle	[45,77] cAMP not increased	[59,76] AC not stimulated	[42,77] PDE inhibition	
Human atrium		[78] AC stimulation and inhibition		
Human	[20] AC stimulation in normal adult heart, [74] No stimulation of AC in adult failing hearts, [74] AC stimulation human fetal heart		[79] AC stimulation, [79] PDE not inhibited	
Rabbit	[76] AC not stimulated in membranes		[76] AC not stimulated in membranes, [80] AC stimulated in nuclei	
Monkey	[81] PDE inhibition [42] AC not stimulated	[42] AC not stimulated	[42] AC not stimulated	
Mouse	[77] PDE inhibition			
Neonatal rat heart	[72] No AC stimulation			
Rat heart	[42,45,82,83] AC stimulation	[45,84] LTCC stimulation [84] cAMP increase [85] AC not stimulated [82] fetal rat AC not stimulated	[86] TnI phosphorylation increased [85] AC stimulated	Augmentation by PDE III [71] or IV [72] [87,88,89,90] cAMP

AC: activity of cardiac adenylyl cyclase, PDE: phosphodiesterase, cAMP: 3′,5′ cyclic adenosine monophosphate, and LTCC: L-type calcium ion channel.

**Table 3:** List of agonist, antagonists, and inhibitory antibodies available for the study of glucagon receptors in vitro and in vivo.

Compound:	Affinity at GR	Organism Cells	Half Life
Antagonist HM15136	[95] Human GR: EC_50_ = 92 pM	CHO, Mice	In mice: 136 h
Antagonist: nonpeptide:2-(4-pyridyl)-5-(4-chlorophenyl)-3-(5-bromo-2-propyloxy-phenyl)pyrrole (L-168,049)	Human GR [96] IC_50_ = 3.7 nM	Mice	
Antagonist: nonpeptide: *N*-[3-cyano-6-(1,1-dimethylpropyl)-4,5,6,7-tetrahydro-1-benzothien-2-yl]-2-ethylbutanamide (SC203972)	[96] Human GR IC_50_ = 181 nM	Mice	
Antagonist, peptide: desHis1-Pro4-glucagon	[97] Human GR IC_50_ = 1 nM	Mice	persistent biological effects
Antagonist, peptide, des-His1-[Glu9]glucagon, GR specific,	[98] Rat GR	HEK	
LY2490921 GR unspecific	[98] Rat GR: 1.3 µM, [98] Human GLP-1-R: 1.2 µM	HEK	
Antagonist, peptide desHis1Glu9(Lys30PAL)-glucagon	[99] GR: 170 pM	Mice, HEK293	persistent biological effects
Antibody REMD2.29	[100] 30 pM		
Glucagon: physiological agonist	[95] Human GR: 800 pM [98] Rat GR: 400 pM [98] Rat GLP-1R: 4.9 nM		[95] 5 min
Sacubitril: inhibitor of glucagon degradation	[101] Inhibition of glucagon metabolism: about 1 nM sacubitrilat		
Anti-sense for GR		[102] Mice, [103] patients T2DM	
Antagonist: Exenadin 9–39	[98] IC_50_: Human GLP-1-R: 17 nM		
Exenadin4: GLP-1-receptor agonist	[98] EC_50_: Human GLP-1-R: 30 pM		
GLP-1	[98] EC_50_: Human GLP-1-R: 65 pM		

GR: glucagon receptor, GLP-1: glucagon-like-protein-1, min: minutes, h = hours, EC_50_ = half maximally stimulatory concentration, IC_50_ = half maximally inhibitory concentration. pM: pico (10^−12^) mole per liter, nM: nano (10^−9^) mole per liter, and µM: micro (10^−6^) mole per liter. HEK: human embryonic kidney 293 cells, CHO: Chinese hamster ovary cells, and T2DM: type 2 diabetes mellitus.

## Abbreviations

AC	adenylyl cyclase
AV-artery	special artery that transports blood into the AV-node
AV-block	failure of conduction of depolarization to pass through the AV-node
AV-node	specialized muscle fibers in the atrioventricular node of the heart
cAMP	3′,5′ cyclic adenosine monophosphate
cGMP	3′,5′ cyclic guanosine monophosphate
Ca^2+^	divalent calcium ion(s)
DG	1,2-diacylglycerol
EC_50_	half maximally stimulatory concentration of a drug
EPAC	cAMP-binding exchange protein
ERK	extracellular signal-activated kinase
Fura2	a drug that shows Ca^2+^-dependent changes in its fluorescence
GLP-1	glucagon-like-peptide-1
GLP-1-R	glucagon-like-peptide-1-receptor
Gi	G-protein that inhibits the activity of AC
Gq	G-protein that stimulates PLC
Gs	G-protein that increases the activity of AC
GR	glucagon receptor
G-protein	GTP-binding protein
GTP	guanosine triphosphate
h	hours
H89	an inhibitor of the activity of PKA
IBMX	3-isobutyl-1-methyl-xanthine, an inhibitor of the activity of PDEs
IC_50_	half maximally inhibitory concentration of a drug
IP3	inositol-1,2,5 trisphosphate
kDa	kilo Dalton (molecular weight of a protein)
KO	knockout, animal with genetic deletion of a gene
LTCC	L-type calcium ion channel
mg	milli (10^−3^) gram
µg	micro (10^−6^) gram
MAP kinase	mitogen activated protein kinase
min	minute(s)
mL	milli liter
mM	milli (10^−3^) molar concentration of drug
µM	micro (10^−6^) molar concentration of drug
Mn^2+^	divalent form of magnesium
mRNA	messenger ribonucleotides
nd	not determined
nM	nano (10^−9^) molar concentration of drug
PCE	positive chronotropic effect (increase the number of heartbeats per minute)
PDE	phosphodiesterase
PIE	positive inotropic effect (increase in force of contraction)
pH	potentia hydrogenii: negative decadic concentration of free protons in an aqueous solution
PKA	cAMP-dependent protein kinase
PLB	phospholamban
PLC	phospholipase C
PPARα	perixosome proliferation-activated receptor alpha
pM	pico (10^−12^) molar concentration of drug
RNAse	ribonucleotide degrading enzyme
SERCA	sarcoplasmic reticular Ca^2+^-ATP(adenosine triphosphate)ase
SGLT2	sodium glucose transporter 2
TnI	inhibitory subunit of troponin
X-ray	Roentgen rays
